# Liver transplantation in the critically ill: a Canadian collaboration

**DOI:** 10.1186/cc11002

**Published:** 2012-03-20

**Authors:** C Karvellas, T Lescot, H Vahidy, P Goldberg, P Chaudhury, P Metrakos, N Kneteman, G Meeberg, M Sharpe, J Ronco, E Renner, E Cook, S Bagshaw

**Affiliations:** 1 University of Alberta, Edmonton, Canada; 2 Hôpital Saint-Antoine, Paris, France; 3 McGill University, Montreal, Canada; 4 University of Western Ontario, London, Canada; 5 University of British Columbia, Vancouver, Canada; 6 University of Toronto, Canada; 7 Harvard School of Public Health, Boston, MA, USA

## Introduction

Critically ill cirrhotic patients awaiting liver transplantation (LT) often receive prioritization for organ allocation. Outcomes in these patients are multifactorial, and identification of patients most likely to benefit is essential. Despite the need for evidence-based allocation criteria based on patient factors and physiology scores, few data currently exist on outcomes. Scoring systems such as MELD and SOFA (Sequential Organ Failure Assessment) are in use, but have not been evaluated in predicting outcome with LT.

## Methods

In a five-center Canadian study (Edmonton, Montreal, Toronto, London and Vancouver), all cirrhotics admitted to the ICU requiring organ support (mechanical ventilation, vasopressors or renal replacement therapy (RRT)) prior to undergoing LT between January 2000 and December 2009 were examined. MELD and SOFA scores were evaluated at ICU admission and the day of LT along with other donor factors.

## Results

A total of 198 cirrhotics (mean age 53 years, 66% male) were reviewed. The most common etiologies were hepatitis C (31%) and alcohol (15%). LT occurred a median time of 29 (5 to 101) days from listing and 5 (3 to 10) days from ICU admission. In total, 88% of patients required vasopressors, 56% received RRT prior and 87% were ventilated prior to LT. The median MELD score was 34 (26 to 39) on ICU admission and 34 (27 to 40) on the day of LT respectively. SOFA scores were 12 (10 to 15) and 13 (10 to 17) on ICU admission and on the day of LT respectively. Comparing patients who were alive (*n *= 166, 84%) versus dead (*n *= 32, 16%) at 90 days, there were no statistically significant differences in MELD score on admission or day of LT (*P *> 0.6 for both). There were also no statistically significant differences between SOFA score on admission or day of LT (*P *> 0.17 for both). Patients alive at 90 days were significantly younger (52 vs. 56 years, *P *= 0.007). Patients over 60 had significantly higher 90-day mortality (27% vs. 13%, *P *= 0.04) and a trend towards increased 1-year mortality (37% vs. 23%, *P *= 0.09). There were no significant differences in donor characteristics (donor age >60, cold ischemia time >8 hours, split graft, donor cerebrovascular event) comparing patients alive at 90 days to nonsurvivors.

## Conclusion

Older critically ill cirrhotics (over 60 years) undergoing liver transplantation have significantly worse post-LT outcomes. MELD and SOFA scores do not appear to predict outcome post LT in this cohort.

